# The experience of sudden loss of a colleague or neighbor following the MH17 plane crash in the Ukraine: a qualitative interview study

**DOI:** 10.1186/s40359-020-0379-8

**Published:** 2020-02-11

**Authors:** C. Joris Yzermans, Christos Baliatsas, Peter G. Van der Velden, Michel L. A. Dückers

**Affiliations:** 1 Nivel – Netherlands Institute for Health Services Research, P.O. Box 1568, 3500 BN Utrecht, The Netherlands; 2 CentERdata, and Tilburg University’s Network on Health and Behavior (Nethlab), Tilburg, The Netherlands; 3 ARQ National Psychotrauma Centre, Diemen, The Netherlands

**Keywords:** Psychosocial impact, Disaster, MH17, Grief, Qualitative

## Abstract

**Background:**

The literature on loss and traumatic grief after disasters provides findings on the impact of losing a partner, child or close friend on partners, parents and friends. However, little attention has been given to the broader everyday social environment of deceased persons. The present study constitutes a qualitative exploration of the impact on colleagues and neighbors following the MH17 airplane disaster in the Ukraine, July 2014.

**Methods:**

Eighteen structured interviews were conducted with eleven colleagues and seven neighbors. The interviews focused on the relation(−ship) with the victim, on the disaster, the first days and weeks hereafter, and the status one and a half years after the crash.

**Results:**

Especially for colleagues and neighbors with an intensive, long-lasting relation and ties based on friendship and trust, the impact of the sudden death was large. The MH17 disaster was considered a special event, different from, for instance, an “ordinary” accident. It was actively covered by the media and a recurrent conversation topic in meetings with other people. In the workplace, employers and less involved colleagues show empathy for a limited period of time, but grief has an expiration date – a moment where it gets more difficult to others or influences productivity. The appreciation for rituals in the workplace or in the neighborhood varies.

**Conclusions:**

The interviews indicate a “hierarchy of bereavement”. People are not part of the typical inner circle, but feel “affected” and experience little social recognition and acknowledgment, particularly in the longer term. As such, colleagues and neighbors may experience loneliness and/or isolation. Generally, there is no need to consult a practitioner, despite the experience of health complaints such as intrusive dreaming and lack of sleep.

## Background

On the 17th of July 2014, flight MH17 crashed in a field in the eastern part of the Ukraine. All 298 passengers and crew members lost their lives on their journey to Kuala Lumpur, Malaysia or further. Among them were 196 people from the Netherlands deriving from communities spread all over the country [1]. The events that followed had a large impact on the affected families and the Dutch society as a whole. It was not an accident. Studies identified a rocket (‘BUK’) attack as the cause, most likely to be initiated by pro-Russian rebels that are involved in an armed conflict with the Ukrainian governmental army [2]. The MH17 disaster rapidly developed into a geopolitical conflict, in which Dutch government strived at clarifying the exact causes. Apart from the inquiry into what happened in the disaster area, questions regarding what the people inside the plane experienced during their final moments, the public attention in the Netherlands focused strongly on the losses incurred by the bereaved families. People lost partners, parents, children, siblings, grandchildren, grandparents, other relatives, friends, community members, colleagues and neighbors. Eventually, many people in the Netherlands personally knew someone who died in the MH17 plane crash.

The government invested in various types of aftercare and expressions of recognition and social acknowledgement of the emotional impact on next of kin. Repeatedly, meetings behind closed doors were organized where families could be informed about the ongoing research into the causes of the plane crash, the repatriation of human remains to the Netherlands, and the identification process. An online information and referral centre (IRC; a website with a public and a private area) was launched a couple of hours after the crash to allow families to consume verified information before it was further disseminated, and where they could also ask questions to different agencies, service providers and experts [3].

In addition, contact with other bereaved families was facilitated and people could sign up for a newsletter of the Dutch Victim Support organization (“Slachtofferhulp Nederland”). In addition to a day of mourning, several commemorations including a national commemoration were organized. In all these activities, the focus was mainly directed at a circle of direct (blood-) relatives left behind: partners, children, parents, siblings, grandparents and grandchildren. Less attention was given to the people falling outside this inner group - people who, strictly speaking, are not next of kin but who coexisted (intensively) with the deceased in daily life, at work or in the neighborhood. Many people spend most hours of the day with colleagues at work, forging special bonds of trust and friendship that are different from those in other relationships [4]. Close colleagues understand people’s ambitions, successes and frustrations better than an outsider: it is a relationship that can often feel closer than with family [5], while relationships with other colleagues are rather superficial and do not go that ‘deep’ [6, 7]. Living near neighbors can also be intense and on average a neighbor knows more than a companion from another town or village.

### Studies on health effects and grief at the workplace

Much is known about possible health effects in the aftermath of disasters on those directly exposed [8–16]: There is a strong body of evidence providing insight into psychological problems such as anxiety, depression, posttraumatic stress disorder (PTSD as well as somatic symptoms and disorders that develop and (usually) disappear as time goes on. However, little literature exists on the effects on health and well-being of colleagues and neighbors of victims of disasters. We found two empirical studies [17, 18], which mainly focus on absence from work after the death of a colleague. We found no literature about health effects on neighbors.

In theoretical articles, the notion of ‘disenfranchised grievance’, introduced by Doka [19], is discussed in particular. This is in fact a rejection of the ‘right to grieve’ and a lack of empathy on the part of the employer, which, among other things, leads to a lack of social support and the inability to participate in rituals. In addition to colleagues and neighbors, according to Doka, something similar happens after the death of friends, ex-partners, mistresses, caregivers, teachers and celebrities, such as Princess Diana and Kurt Cobain. Eyesemitan [17] states that disenfranchised grievance means that in the workplace the loss or mourning is not openly acknowledged, not socially sanctioned nor publicly shared and labels the situation on the work floor as ‘stifled grief’. Hazelton added a broader dimension to the problem [20]: “In our death-denying culture, grief is a personal thing not shared with others. We cannot leave grief at the doorstep of our workplaces. We cannot deny that symptoms of grievance create stress that affects people and, therefore, their workplace”. Finally, Albert [21] points out that a subgroup of mourners has arisen whose legitimacy is not recognized and whose needs are not seen. These people do not have access to social ceremonies and memorial activities.

The aim of this research is to gain insight into how colleagues at work and neighbors experience sudden loss of, in this case, a victim of the MH17 disaster. How do they grieve, at home and at work, and what possible effects are there on their health and well-being?

## Methods

The present qualitative study employed qualitative description [22–24] as primary research approach. The aim of such approach is to provide a straight-forward description of phenomena in everyday language [22]. Additionally, a phenomenological overtone was incorporated (Sandelowski, 2010). Structured individual (face-to-face) interviews took place 15–18 months after the air disaster (November 2015–February 2016), using a structured checklist that was developed for the interviews of both colleagues and neighbors (see Additional file 1).

### Checklist development

The current version of the structured interview was used for the first time in the present study since there was no available/previously published checklist in this field that would be directly applicable to this context. The included questions do not form an instrument since the purpose of the present study was not a quantitative assessment or diagnostically-oriented evaluation. Questions were derived from the existing literature [25] and partly based on questions that were asked in the context of concurrent research among relatives (with blood ties) of the victims of the MH17-disaster in other stages/waves of the project [26, 27], such as the ones included in the Traumatic Grief Inventory [28]. Questions were adapted to the setting of relevance, taking into account the nature of the relationship between victims and responders and the background and context of the disaster incident: “how were the victims commemorated in the workplace” versus “how were the victims in the neighborhood / village / city commemorated?”

### Participants

Participants were selected based on purposive sampling. Since the names of colleagues and neighbors of the victims of the MH17 disaster are not registered in a formal database, we approached people who were mentioned in newspaper reports and interviews (tracked via websites and telephone directories) with the request to participate in the study. Prior to providing consent (verbally), participants were informed that the interview data would only be reported at an aggregated group level and would not be traceable to individual persons. Biographical data/personal information is therefore not collected nor reported in this article, apart from gender. According to the Dutch Medical Research Involving Human Subjects Act (WMO) the present study did not require ethical approval. The present paper follows the criteria for reporting qualitative research (COREQ) [29], when applicable.

Of the 20 invited, 18 accepted the invitation (11 colleagues and 7 neighbors, 9 women and 9 men; except for one responder who was 18 years old, all participants were adults older than 30 years old). One of the participants was both a neighbor and a colleague; since this respondent worked at a completely different department in the company, this person was counted as a ‘neighbor’. We searched as much as possible for duos: two colleagues or neighbors of the same MH17 passenger, because there could be few participants and we wanted to limit heterogeneity. Saturation was reached when it was not possible to obtain additional new information and recruit more participants.

### Procedure

All 11 colleagues were interviewed by the same, experienced interviewer (MLAD) at the participant’s workplace or at his or her home. A second interviewer conducted all neighbor interviews. There was no prior relationship between the participants and the interviewers and no personal information of the interviewers was known besides affiliation and expertise. The aim of the research (as described earlier) was presented to the interviewees at the start. Interviews were carried out once, and no other person was present besides the participants and researcher. The conversations were digitally (audio) recorded (in combination with field notes) and transcribed by a professional agency. Sample-wise text and tape were checked by the first author. One of the interviews involved two colleagues and one two neighbors at the same time, in which the answers per participant were noted, recorded and analysed. In this case the MH17 casualties were two married colleagues employed at the same company. To ensure confidentiality, each participant was assigned a unique identifying number.

### Analysis

To analyse the transcripts, the first author made use of a data extraction form that followed the order of the checklist items. In order to remain closer to the data, the clustering of the items into themes was based on the extracted information. More specifically, transcripts were analysed on the basis of the strategy of content analysis in qualitative descriptions [23, 30], entailing data coding from interviews, documentation of insights/reflections on the data, identification of similar phrases, patterns, themes and common characteristics in the data, consensus within the research group regarding generalizations for the data and investigation of these generalizations on the basis of existing knowledge/literature.

To maximize confirmability, researchers tried to remain as neutral as possible during content analysis/interpretation. To enhance study quality and rigor, after the formulation of the thematic descriptions, participants were asked to verify the intended meaning (the so-called respondent validation/“member checking”) [23, 31].

## Results

Interviews lasted between approximately 30 to 90 min with a mean interview duration of 48 min. Phrases and words related to the experience of sudden loss of a colleague or neighbor were underlined and extracted as primary codes (see “descriptive coding tree” in Appendix), which were categorized to 11 themes: These were the following: “Relationship to victim”, “The day of the disaster”, “The first weeks”, “Identification”, “Eighteen months after the incident”, “Commemoration: the first period”, “Commemoration: Eighteen months after the incident”, “Practical consequences”, (main themes) and “Unlike a car accident”, “Media attention”, “Attending national commemoration event” (minor/supplementary themes). The derived themes were critically discussed within the research team until agreement was reached, with only a few adjustments required.

In the text literal quotations are always displayed in italic; several comments from the same respondent are separated by a ‘/’, while a ‘;’ separates the different respondents. If the quotation were traceable, it has been adapted by the authors. Table 1 summarizes participants’ perspectives on the disaster the first weeks after the incident.

**Table 1:** Participants’ perspectives on the disaster the first weeks after the incident

Respondent ID	Role	Gender	Citation
C1	Colleague	Female	*“You think that is simply not possible. It is not a car accident that you often hear about, so that’s where denial comes from.”*
C2	Colleague	Male	*“[I think about my colleague] every single day. It can hardly be otherwise … I still work in the same building. His enthusiasm and drive can still be felt here.”*
C3	Colleague	Female	*“[I felt] as if I had swallowed a piece of stone, really. I cried a lot. […]* *I’ve been thinking all the time about how strange life is and what gets lost and how that affects our understanding of the world.”*
C4	Colleague	Female	*“The first few weeks, I think when I was at work, I received a certain kind of attention, because I had known [the victim] for so long. I was just very sad, although I don’t feel that this is the right word, it was actually more than that. I could function though. I slept very badly, I had nightmares all the time.”*
C5	Colleague	Male	*“I am not really an emotional person, but if you lose someone you have a good relationship with, then you just get hit, it is as simple as that.”*
C6	Colleague	Female	*“(I felt) dazed. I was like: this is not possible, because she would have normally travelled a week later [...] You are defeated, you just can’t get over it. You are still hoping that she was not on board and you are looking at your phone [...] it was unreal and it still is actually.”*
C7	Colleague	Male	*“It was crazy, unbelievable and sometimes hard to realize, because nothing preceded the incident, it all happened suddenly at once, so you don’t actually believe it. […]* *… you are trying to imagine what happened. [You wonder:] Did they notice anything?...”*
C8	Colleague	Female	*“[I] certainly [felt] disbelief in the beginning. That evening and night I was also thinking: she wasn’t on board. You don’t want to know that at all, so you push these thoughts away. Then the next day messages were coming from everywhere [...] What you feel then is sadness and also anger (because of) the way (they died), because I thought that was also a big deal [….].* *This was not supposed to happen.”*
C9	Colleague	Male	“*“… we think of the family, friends and colleagues” [...] I believe this has an enormous significance. I think that if you mention something like that during a commemorative speech, when everyone desperately seeks for sympathy and grip, that’s really something therapeutic.”*
C10	Colleague	Male	*“It’s a weird feeling that you cannot define. [During that moment] so many things happen. You don’t know yet what, how. Little by little you get some information, something to hear. You are not family but you are very closely involved … [...] My wife sometimes says: “It is anger that I hear in your voice”, because you had built something together. [...] It feels like family, that special bond you have.”*
C11	Colleague	Male	*“(The first few weeks at work) there was less room for emotions and more energy for work. [...] I noticed that at the moment you are talking about emotions, these become suppressed and (the need to) work comes forth; but when, for example, we were at his house [...], then the emotions hit us twice as hard. I did notice that.”*
N1	Neighbor	Male	*“I actually had to study, but I just couldn’t.” [...] it is about someone you know that was there, it could have been my parents or a friend of mine or something. [...] When I first heard it, I got really angry (at the perpetrators), but once the funeral was over, the anger was not there anymore.”*
N2	Neighbor	Male	*“We were very sad the first few days, I also couldn’t sleep well, but it’s not that I could not function anymore*” *[...] How should I express that? It’s the fact that they were the next-door neighbors, that hits the hardest. If it was someone who lived farther away or in another village, it wouldn’t be the same.”*
N3	Neighbor	Female	*“It was just murder actually. That has a different impact compared to being in a car accident. This makes you angry. [...] [if it was just an accident] it might have been easier to make peace with it.”*
N4	Neighbor	Female	*“I still find bizarre that when I first saw it on television, I reacted immediately like: this feels really wrong. [...] ....even though I didn’t really know which day she was flying and in the beginning I did not realize that at all. I really had to force my husband to go have a look [...]. I think a lot about [the victim], actually more than when she was alive.”*
N5	Neighbor	Female	*“No matter how crazy it may sound, a car accident is part of ​​your daily life. You don’t want it, but somehow it is part of it. This [the disaster] was so out of place. This is absolutely not how I perceived safety and security.”*
N6	Neighbor	Male	*“I was looking for information and when it was on the news, I was sitting in front of the TV and I also followed the national commemoration and every day when it was in the news I was following it. [...] I wanted to know everything that happened, but it’s not that it controlled my life.”*
N7	Neighbor	Female	*“[It was stressful] that they kept coming … the journalists … and that is something I want to impart: please, when something like this happens, journalists should be banned from coming here …*”

### Main themes

#### Relationship to victim

The interviews started with four items, addressing the degree/intensity of the relationship with the victim. Seven of the 11 colleagues knew the deceased for a long time, had a close relationship (in four cases described as a ‘bond of trust’) and also worked together for a very long time in the same company. That is not to say that they visited each other at home, outside working hours (this was the case three times). Qualifications about the intensity of the relationship include: *familiar and sociable; very dear friend / we shared love and suffering / leading the company was like a marriage; same age / same sense of humour; worked together with his father; close relationship / a lot of traveling together*.

The four colleagues who knew the deceased for a shorter time (3–5 years) had a less intensive relationship: *good professional contact; we were always talking about this and that; deep respect; good relationship*. The neighbors described their relationship as *good / you could always call on them; not real friends, but you know a lot about each other / we did a lot together*.

#### The day of the disaster

All interviewees heard on the day that the plane had crashed, often via media (television, radio, Facebook) or through family and friends, and in some cases via work or a neighbor. For almost everyone it was clear the next morning that the colleague or neighbor had been on the plane. In one case it was not immediately clear, which led to denial and confusion. The immediate effects on the interviewees of hearing the sudden death of colleagues or neighbors were diverse, as recalled and expressed one and a half years later: Emotionally: *denial; terrible crying; as if I had swallowed a stone, grotesque / unimaginable; it hit me; disbelief / sadness / anger; I try to picture/imagine it / violent shocking; empty / unreal / gripping, blind panic / raw sadness, attack on your sense of security / fear.* In relation to work: *‘just’ continue; arranging things; making a lot of calls; as a family you take a week off, as a colleague you just go to work; survival or survival mode; emotion transformed into hard work but privately it kept coming.*

#### The first weeks

Most of the colleagues and some neighbors focused themselves on their work after the disaster, because that was necessary and also “to have a form of distraction”. Different terms were used, such as: *survival mode; carer role (made my own grief smaller); in a daze*. Three colleagues were also bothered by the daily confrontation that their colleague had died in the disaster. One of the colleagues therefore stayed at home a lot for the first few months. Another did not feel that her grief was acknowledged. Two colleagues, who did not know the victim for too long and had a less intense relationship, did not experience any major problems in the first weeks. In dealing with sadness almost all colleagues and neighbors indicated that they had felt sad in the first few months, had sleep problems and frequent dreams (*slept 3–4 h a night during the first half year; repeated nightmares how he fell from the sky; I was mainly awake; besides dreaming I had many associations, especially with music*). In addition to grief and sleep problems other reactions were mentioned, like: *very much upset; irritated / angry* (mentioned by 6 interviewees)*, I was brought out of balance; headaches; exhaustion; back pain; bleak; had to talk about it all the time*. Four people consulted their general practitioner (GP), one of them was prescribed antidepressants. One of the neighbors had a one-off contact with an employee of victim support (*very supportive*) and another neighbor consulted a psychologist.

#### Identification

Because many of the victims were part of a family, and shared the same DNA, it was not always clear when persons were (totally) identified. There were cases in which funerals were only organized when all members of a family were identified. As far as the memory of the interviewees allowed an estimate of the time the identification lasted, periods of about two to more than six months were mentioned. The news that the neighbor or colleague was identified, generally came from the family or from a manager from work; however, quite often it became known days or weeks later or heard by chance. Five interviewees had direct contact with the surviving family, mostly in the first period after the incident; later there was less or no contact at all. Colleagues and neighbors were poorly informed about the identification process which resulted in dissatisfaction and discomfort: *no, you do not count; you keep grinding over those bodies and you are not being acknowledged as a grieving person; why did not they appoint one of us as confidant to the family?; and then you also get questions about it from customers; a neighborhood committee was set up, but they did not keep us informed; we have protested against that; it was often confusing: when you though it was over, then a finger or a toe was found in the fields in Ukraine.*

In one case, family, friends / acquaintances, neighbors and colleagues were members of a social media group (WhatsApp), so information came through quickly. Colleagues and neighbors might have also sought for information themselves. Five of them deliberately did not seek information (*I don’t like publicity and sentimentality*), while others actively looked for specific information, e.g. particular aspects of identification. As mentioned earlier, lack of information was a problem: *we were roommates and friends and we heard the news much later and* via *others, that is lack of recognition of our position; in the church we also had to sit behind the management*. Compared to the colleagues, all the neighbors actively searched for information: *I wanted all details; I signed up for the victim support newsletter; searched for their pictures on the internet*. None of the interviewees ever visited the public part of the IRC website that was established after the disaster for relatives and caregivers.

#### Eighteen months after the incident

One and a half years after the disaster, most effects on health and well-being have vanished. Four colleagues said they were feeling tired and two of them still angry (accompanied by powerlessness), one of them still felt guilty, while another indicated having less control over her life. With the exception of two colleagues and two neighbors most respondents still missed the deceased, one more than the other: *he was my touchstone; I often think of him, but now more quietly; it feels like I have lost a family member; he was my sparring partner and we shared a mission; something is broken in my life*. For people with more intensive contact with the victim, it took at least a year before they could “make peace with it” *her workplace was empty for a long time; the business was recovered faster than the emotions*.

#### Commemoration in the workplace / neighborhood: the first period

With one exception (*they did not organize anything, I’m still angry about that*), commemoration meetings were held shortly after the news, and in some cases ex-colleagues were also invited. All colleagues attended the funeral. Photos of the victim were placed at the entrance or in a common room where a ‘private spot’ was arranged, with flowers and candles. At three workplaces a special Facebook page was created and there were initiatives, such as planting a tree, making a memorial booklet (for family and for regular customers) and organizing a hike.

In the neighborhoods the nature of commemoration activities varied: in one case flowers were deposited at the house (*it looked just like Lourdes*), flags were flown half-staff. In other neighborhoods nothing happened, or in some cases schools and sport clubs also organized commemorations in September, after the holidays ended. In one of the neighborhoods a committee was set up, which, among other things, planned the creation of a monument. When a whole family had disappeared, the number of activities was larger.

#### Commemoration in the workplace / neighborhood: eighteen months after the incident

At a certain stage, some of the commemorative actions carried out in the beginning have to be reconsidered and even ended. Colleagues and neighbors described how dilemmas emerged when the photo was removed from a workplace (for one person that is a permanent memory, while another person no longer wants to be confronted with it), when the employee’s position (depending on his/her function) is succeeded, or when the neighbor’s house gets a new resident. Especially when the colleague has a specific expertise or a position high in the hierarchy, a replacement is not easily arranged, which is accompanied by a potential increase in the workload of other colleagues. Colleagues quickly picked up normal routine as efficiently as possible and aspects of grief were mainly kept and processed privately. The main assignment awaiting the managers was to understand, recognize and respect feelings of grief and loss, and at the same time prevent these feelings from affecting the functioning of the staff, as well as their productivity. Compassion was mainly expressed in giving days off and a temporary reduction of the workload. As a result, there was little room for collective grief and commemoration in the mid and longer term. Several colleagues indicated that they were still (seriously) missing their lost co-worker, one and a half years later, which is logically related to the nature/intensity of their relationship to the victim (see also theme “relationship to victim”).

The neighbors got over the loss comparatively faster. The arrival of new neighbors facilitates the transition to a new situation. Two interviewed neighbors indicated that they found it positive that new neighbors were coming. One of the neighbors was determined not to build up an intimate relationship with the new neighbors again. The social media (WhatsApp) group mentioned earlier was closed after one year.

### Practical consequences

Almost all colleagues encountered practical problems. Privacy issues made it impossible to gain access into the victim’s computer. This complicated the continuation of work and contact with clients. Duties of the victim had to be taken over soon, in an ad hoc manner, without preparation or time to prepare: *her position was not filled; everything is now on my plate, which breaks down her work.* The commercial settlement with heirs, tax authorities, banks (the authority to sign, for example) was a stressor on the longer term. Successors had a difficult start as comparison with their predecessor was obvious: *the successor did all kinds of things differently; that is not how X would have wanted it; a dilemma emerged between emotions and business*.

It was common for the neighbors to maintain the garden of the neighboring house that was secured by the police. It was confronting that the car of the neighbor kept standing on the driveway. One man who lost his wife in the disaster received emotional support from his neighbors and also help regarding practical issues.

### Minor themes

#### Unlike a car accident

The interviewees were asked whether the emotional impact would have been different when the colleague or neighbor would have been involved in a fatal car accident. Most of them considered this a large difference*: there was uncertainty for a long time on the identification, but also about the cause and whether they have suffered; this was often on the news: no escape from it, everywhere you go, it becomes extreme / many people who have an opinion about it; an accident is a more accepted risk this was murder: it makes you angry / feeling unsafe now*. In the experience of the respondents there are big differences compared to other possible causes of death. To what extent this has an influence on mourning and how it might have affected health and well-being cannot be determined with certainty based on the interviews.

#### Media attention

Some colleagues read newspaper reports and watched television from the beginning, particularly about the return of the bodies to Eindhoven Airport and the ceremonial transfer to the location of the identifications. Others tried desperately to shut themselves off, but there was hardly any escape. Seeing pictures sometimes had a healing effect (*it puts the loss in perspective, it helps with the grieving process*), while on the other hand it nourishes the imagination. According to one of the colleagues, the press is only occupied with sensation and blame games: this bothered her. People who lost a neighbor family were repeatedly approached by television journalists in the street, up to a year after the disaster. People within the neighborhood agreed to ignore the media and keep the curtains closed. One of the neighbors pinpointed: *I follow everything and it touches me every time.*

#### National commemoration event

Finally, the participants were asked whether they would have liked to attend the National Commemoration organized for direct next of kin. The vast majority of the respondents (with the exception of one neighbor) gave a negative answer. Such a massive gathering is not considered appropriate for everyone. However, in several cases it was indicated that it would have been good if there was more recognition for the position and grief of the colleagues. Two neighbors were pleased that they received a letter from the municipality with a reference to victim support and a telephone number.

## Discussion

### Experience of sudden loss of a colleague or neighbor

The interviews illustrate that the impact on colleagues and neighbors was generally large. Formally, one is not “affected”, in practice it is considerably more complex. Especially for colleagues and neighbors with intensive, long-term contact, accompanied by friendship or a relationship of trust, the impact was and is large. The study reveals that after the MH17 disaster there was also a subgroup of mourners in a second or third circle around the victim, which receives little attention from researchers, governments, the royal family and media. Nonetheless, their grief is intense and the sharp edges only disappear after more than a year. Social support and acknowledgement of the emotions are not always self-evident. At work, employers and less involved colleagues have sympathy for a limited time, but expressions of mourning have an expiration date, a time when things become difficult for others [5], or for example when it affects productivity (matching concepts such as ‘disenfranchised’ or ‘stifled’ grievance; see the paragraph on the literature). This limitation therefore also has consequences for private life, after office hours and in the weekend. A disruption arises between work and private compartments [7]. Additionally, co-workers are confronted with an increased workload to replace the absentee, to serve customers, and problems ranging from ethical (access to computers and mail, sign authority) to practical (changed tasks, schedules, appointments, unfamiliarity with specific expertise or with the status of processes).

### Emotions at home and the workplace and confrontation of public events

Colleagues and neighbors experienced personal effects of a disaster that received a great deal of public attention. Apart from grief and the feeling of being an outsider, they expressed several emotions, especially anger. This anger was connected to the colleague’s fate, the apparent coincidence, the probable Russian involvement, and the difficulties in bringing the bodies of the victims back to the Netherlands. However, the interviews showed another type of anger or resentment following from the fact that co-workers’ own work package was expanded with additional tasks, and – in case of the neighbors – the exposure to ever-recurring television teams and the sea of flowers or mini-monuments in front of the neighboring house. Other emotions reported were shame, guilt, fear and confrontation with one’s own mortality. Besides, for some of the interviewees the MH17-related losses were not the only large life events they experienced in the one and a half years after the disaster.

The appreciation for rituals in the workplace or in the neighborhood varies. For one person, a photo of the lost colleague with flowers and candles gives solace, for another it feels like a constant confrontation. The thought that it is important to mark that someone has lived and that you worked together for a while is not disputed [32], but at a certain point the question arises: when should the photo be removed? Nobody wants to be disrespectful, and the removal of the photo can coincide with ‘the expiration date’ of public expressions of grief in the workplace. Another confrontational moment is when the function of the missing colleague is taken over by a definitive replacement. In the interviews various rituals were mentioned in the workplace, but their valuation was, as written earlier, individually determined. In the case of two colleagues, nothing was done by the company and that was not appreciated by the participants. Three other colleagues kept as much distance as possible. Burton et al. [33] argue that managers should be visible and set the tone for resilience and recovery, where resilience is based on strength and not on pathology [34]. Frequent communication is essential in this. In some instance the neighborhood, the community or the city did not organize commemoration activities (“*we are not like that*”), while in other places several rituals were carried out and, for example, a monument was erected by the neighbors themselves. Most neighbors had trouble with confrontations: the aforementioned sea of flowers and the pushy television crews, the sealed house, the car in the driveway, the proliferating plants in the garden, and people who openly express their grief. Most neighbors more or less forgot about it after the arrival of new neighbors.

It is striking that, with one exception, no negative aspects were mentioned about the deceased. Social desirability can play a role in this.

### Hierarchy of bereavement

Maercker and Muller [35] defined social acknowledgement as “a victim’s experience of positive reactions from society that show appreciation for the victim’s unique state and acknowledge the victim’s current difficult situation. The term social here not only includes the closest social network of a victim (e.g., family, friends) but also significant persons (e.g., local authorities, clergy), groups (e.g., at the workplace, fellow citizens), and impersonal expression of opinions (e.g., media) about the experiences of the victims or survivors”. This theme of social acknowledgement and recognition is addressed in a majority of the interviews. Interviewees provide examples of feeling excluded or not taken seriously, especially when it comes to obtaining information. Colleagues and to a lesser extent the neighbors have no, or only short-term contact with family and relatives. As a result, in the case of the MH17 disaster, they feel ill-informed, especially around the identification, but also about the on-going inquiry who is to blame. The perceived lack of recognition comes on top of an intense sadness that is difficult to express and not always understood or valued by the environment.

All in all, the interviews might indicate a “hierarchy of bereavement”. Colleagues and neighbors are affected by the loss of people who played a role in their daily lives, and the consequences of that loss, but the impact is not commonly recognized and respected by others. As such, colleagues and neighbors can feel alone and isolated. Generally, there is no need to consult a practitioner like a GP or psychologist, despite experienced health complaints such as intrusive dreaming and lack of sleep.

### Practical implications

The MH17 air disaster was not an average disaster, due to the bizarre cause and long aftermath that has not yet ended. The findings reported above, however, have in our view validity for future disasters and serious incidents. Especially for people with a longer, more intensive bond with a victim. This group is affected and could benefit from a better provision of information and social support to reduce the chance of feeling being left alone. Looking back on the findings, we believe that some practical measures could be taken for colleagues and neighbors on behalf of future disasters. They could, to a certain extend be included (or at least mentioned) in the supportive activities and measures as mentioned in the introduction (e.g. addressed in public speeches, pages on support websites). One of the neighbors, for instance, was a member of a social messaging group (WhatsApp) in which information about identification, repatriation and funeral was shared. On behalf of the workplace one person (not necessarily the manager/director), could keep in touch with the family, so that the delay in sharing information is minimized and information is not by chance communicated via others. Moreover, although governments and media primarily focus on family (and sometimes on friends), there should be a small effort to pay attention to or address the wider circles around the victim. In the case of larger disasters it is impossible to capture the complete networks of victims but colleagues and neighbors are obvious groups to include in communication strategies and proportionate types of practical and psychosocial support.

### Strengths and limitations

When considering the implications of this study, it is necessary to recognize the strengths and limitations of qualitative approaches. To the best of our knowledge, this is the first qualitative study to explore sudden loss in the aftermath of a disaster from the perspective of both colleagues and neighbors of the victims. We included a relatively homogeneous sample with a well-balanced representation of gender as well as colleagues and neighbors.

We used purposive sampling, selecting participants based on relevance to the study purpose as well as availability (considering that colleagues and neighbors of disaster victims is a very specific group that is often hard to reach, given the context and circumstances). Given that a complete list of colleagues and neighbors of the victims of the MH17 disaster was not registered / available, we approached people who were mentioned in media reports and interviews, in collaboration with the Victim Support Fund. This introduces the risk that the views of several potentially relevant participants were not covered. Nevertheless, there is no validated means of objectively establishing saturation, while our sample size seems to be sufficient for the current study approach as prior investigations suggest [36–38]. The fact that, for practical reasons, one of the interviews was conducted for two colleagues and one for two neighbors could potentially introduce some bias. Finally, qualitative descriptions follow a pragmatic approach; as Neergaard et al. [23] pinpoint, no theoretical strings are attached during the analysis. The advantage here is that the analysis follows closely the perspective of the interviewees, but at the same time the subjective element is, to some extent, stronger, due to researcher’s perceptions and/or dispositions. However, critical discussions within the research group during the analytical process and employment of strategies such as “member checking” were used as strategies to cope with this issue.

## Conclusions

The present qualitative study was based on a series of interviews with colleagues at work and neighbors who experienced the sudden death of a victim of the MH17 disaster in July 2014. The interviews did not focus on the most common next of kin but emphasized on the broader everyday social environment of deceased persons. In this expanded outer ring we observed the impact of the disaster on a fairly “forgotten” group of people in their work setting and at home.

Apart from verifying a perceived “hierarchy of bereavement”, reflected in the experience of a lack of social recognition, acknowledgment, and support, we gained a better insight into the emotions, coping styles, and health effects of colleagues and neighbors. The fact that the participants’ health care utilization was low, is no justification to ignore the considerable impact a disaster can have on people in local communities and organizations.

### Supplementary information

**Additional file 1.** (Checklist): Structured interview checklist used in the study

## Data Availability

The dataset used in the current study is available from the corresponding author on reasonable request and with permission of the involved research consortium.

## Appendix

**Table:** Descriptive coding tree from the findings of the investigated themes

Relationship to victim	The day of the disaster	The first weeks	Identification	18 months after the incident	Commemoration: The first period	Commemoration: 18 months after the incident	Practical consequences	Unlike a car accident	Media attention	Attending national commemoration event
Close	Disbelief	Focus on work /distraction	Informed by family or manager	Recovering slowly	Activities varied	Missing coworkers	Work continuation	Unfair	Hard to escape	Not attending
Bond	Sadness	Sleep problems	Avoidance	Missing the victim	Flowers and candles	Compassion	Privacy issues	Uncertainty	Excessive	Need to be acknowledged / recognized
Trust	Denial	Headaches	Searching for information		Memorial	Dilemmas	Confronting what the victims left behind	Hard to understand	Stressful	
Intensity varies	Anger	Pain	Poorly informed		Meetings	Workload reduction		Extreme	Blame games	
Friend	Shock	Feeling sad				Days off				
		Health care utilization/ medication				Avoidance				

## Abbreviations

GP: General Practitioner
IRC: Online Information and Referral Centre
