# Supplementary material for: The experience of sudden loss of a colleague or neighbor following the MH17 plane crash in the Ukraine: a qualitative interview study
Source: BMC Psychol. 2020 Feb 11;8:16. doi: 10.1186/s40359-020-0379-8 (PMC7014754; doi:10.1186/s40359-020-0379-8)
Supplement: Supplementary file 1 — Additional file 1. (Checklist): Structured interview checklist used in the study [file 40359_2020_379_MOESM1_ESM.docx]

**Structured interview checklist.**

*Brief introduction: Provide information on the background and objective of this interview, its context, funding source and the nature of the final report (findings at group level and therefore anonymous). Make clear how the researchers found his / her name.*

Questions:

- Relation with the victim (length and intensity of relationship, meet each other outside office hours / privately at home, special bond, because ..., cooperation);
- How did you hear about the disaster and by whom? Was it immediately clear colleague / neighbour was on board? If not, when was that clear?
- What was the effect of sudden death on you (disbelief, anger, sadness). Are you missing him / her / them? Because ...
- Does it make a difference that the colleague / neighbour died this way, without saying goodbye, and disappeared under these bizarre circumstances, compared to, for example, if he or she was involved in a car accident?
- How quickly was the loss certain / colleague or neighbour identified? How did you hear about it? Were you updated on new developments or findings? Was there contact with the family or relatives of the victim?
- Since the plane crash took place in the summer: did you go on a holiday in July or August (before, during or after the plane crash).
- How did you get through the first weeks? Did you have dreams / nightmares, and physical or psychological health problems?
- What has been done at work / in the neighbourhood in the sense of commemorating or memorizing?
- What was the reaction in the first week and first few months: was there a regular discussion about the loss of the colleague/neighbour, were memories shared with colleagues / neighbours? After how long did people go back to the normal routine? How is that today, more than 1 year ago?
- When, in the past year, was his or her death been given a place? How many times a week or a day do you still think of him or her? How would you describe your mourning reactions? Do you still have dreams / nightmares or health problems?
- During the past year, did you receive treatment from a general practitioner, psychologist or social worker for a complaint that could possibly have been related to the death of your colleague / neighbour? Frequency of contact? Have you taken different or more medication since the disaster?
- Is it difficult for you that there is so much media attention, and that you are always reminded of it? Do you read newspaper reports about the MH17 disaster and watch television programs about it?
- Would you like to attend a national commemoration, as organized for the direct next of kin?
- How did you get information? Was it given to you or did you look for it yourself? If given, by whom?
- Have you ever looked at the IRC website (online information and referral centre for the bereaved)?
- Did you encounter practical problems? Did you work with him or her as a colleague; if so, what did his sudden death mean? Were you as a neighbour confronted with practical matters (home, garden, shared possessions)?
